# A Novel HIV Vaccine Adjuvanted by IC31 Induces Robust and Persistent Humoral and Cellular Immunity

**DOI:** 10.1371/journal.pone.0042163

**Published:** 2012-07-25

**Authors:** Laura Pattacini, Gregory J. Mize, Jessica B. Graham, Tayler R. Fluharty, Tisha M. Graham, Karen Lingnau, Benjamin Wizel, Beatriz Perdiguero, Mariano Esteban, Giuseppe Pantaleo, Mingchao Shen, Gregory A. Spies, M. Juliana McElrath, Jennifer M. Lund

**Affiliations:** 1 Vaccine and Infectious Disease Division, Fred Hutchinson Cancer Research Center, Seattle, Washington, United States of America; 2 Intercell AG, Vienna, Austria; 3 Centro Nacional de Biotecnologia, Consejo Superior de Investigaciones Científicas, Madrid, Spain; 4 Division of Immunology and Allergy, Department of Medicine and Swiss Vaccine Research Institute, Lausanne University Hospital, Lausanne, Switzerland; 5 Department of Global Health, University of Washington, Seattle, Washington, United States of America; University of Pittsburgh Center for Vaccine Research, United States of America

## Abstract

The HIV vaccine strategy that, to date, generated immune protection consisted of a prime-boost regimen using a canarypox vector and an HIV envelope protein with alum, as shown in the RV144 trial. Since the efficacy was weak, and previous HIV vaccine trials designed to generate antibody responses failed, we hypothesized that generation of T cell responses would result in improved protection. Thus, we tested the immunogenicity of a similar envelope-based vaccine using a mouse model, with two modifications: a clade C CN54gp140 HIV envelope protein was adjuvanted by the TLR9 agonist IC31®, and the viral vector was the vaccinia strain NYVAC-CN54 expressing HIV envelope gp120. The use of IC31® facilitated immunoglobulin isotype switching, leading to the production of Env-specific IgG2a, as compared to protein with alum alone. Boosting with NYVAC-CN54 resulted in the generation of more robust Th1 T cell responses. Moreover, gp140 prime with IC31® and alum followed by NYVAC-CN54 boost resulted in the formation and persistence of central and effector memory populations in the spleen and an effector memory population in the gut. Our data suggest that this regimen is promising and could improve the protection rate by eliciting strong and long-lasting humoral and cellular immune responses.

## Introduction

Human immunodeficiency virus (HIV) is responsible for nearly two million deaths annually, and although the overall incidence appears to have stabilized, the epidemic continues to spread (WHO, 2011). A vaccine represents the best possibility for eradication of the virus, but despite unprecedented efforts, an effective vaccine has not yet been developed.

Two recent vaccine efficacy trials provide clues to potential components that can contribute to protective immunity against HIV. Specifically, the Step study used a mixture of recombinant adenovirus serotype 5 (Ad5) vectors expressing HIV-1 proteins [1], [2]. That study terminated early after interim analyses demonstrated that the vaccine neither prevented infection nor lowered viral load, and perhaps had the adverse effect of increasing HIV acquisition in subjects with preexisting Ad5 neutralizing antibodies. Though the reasons for this remain unclear, follow up investigations indicated that adenovirus-specific CD4+ T cells might have impacted the availability of potential HIV target cells [3], [4]. In contrast, the RV144 trial was the first ever to demonstrate modest protection from HIV infection [5]. That trial used an ALVAC-HIV prime in combination with a VaxGen AIDSVAX bivalent gp120 clade B/E protein boost. The regimen induced mostly humoral and low-level CD4+ T-cell responses, supporting the hypothesis that balancing both arms of the immune response will induce improved protection.

New York vaccinia virus (NYVAC) vector is a highly attenuated Copenhagen virus strain capable of inducing humoral and T-cell responses [6], [7]. NYVAC-CN54 encodes cell-released HIV-1 Env gp120 and Gag/Pol/Nef, an intracellular polyprotein harboring cytotoxic T lymphocyte epitopes [8]. Preclinical studies in mice demonstrated that this vector, when used as a boost after a DNA prime, induces HIV-specific CD8+ T-cell responses and IgG production [8], [9]. In monkeys, a similar NYVAC vector expressing HIV gp120 and SIV Gag/Pol/Nef induced CD4+ and CD8+ T cells and antibodies to Env, with protection following SHIV89.6p challenge [10]. In a phase I clinical trial NYVAC-CN54 induced a robust immune response, in particular Env-specific IFN-γ production by CD4+ and CD8+ T cells [11]. Additionally, vaccinia viruses are advantageous vaccine vectors because pre-existing immunity at the population level is restricted to aged groups, since smallpox vaccination was terminated in the mid-1970s. Even in individuals with pre-existing immunity, smallpox-specific T cells are less frequent in the mucosal tissues of healthy volunteers than adenovirus-specific T cells [12], suggesting that use of this vector may avoid problems encountered in the Step study. Finally, vaccinia viruses are capable of activating innate immune responses through TLR-dependent pathways [13], [14]. TLR activation is recognized as a key component in several vaccines, including the yellow fever vaccine, the activity of which seems largely to be due to the generation of Th1-inducing mature DCs [15]. Such findings have opened a new field of research on novel adjuvants, many of which are TLR ligands, to mimic the pathogen-associated molecular patterns recognized during an encounter with a natural pathogen.

Thus far, eleven TLRs have been identified in mice. Among them, TLR3, TLR7, TLR8 and TLR9 recognize nucleic acids; in particular, TLR9 recognizes CpG motif-containing DNA sequences. Several CpG oligodeoxynucleotide (CpG ODN) formulations have already been used as adjuvants in vaccine studies for infectious diseases, including HIV [16], [17]. In this regard, CpG ODN was shown to improve the humoral and cellular responses in a vaccination regimen consisting of Gag protein prime and adenovirus boost, both in mice [18] and in a primate model [19]. The recently developed compound IC31 consists of a combination of ODN1a, a TLR9 ligand, and the antimicrobial peptide KLKL_5_KLK, which contributes to the stabilization of ODN1a and to depot formation [20]. This combination was shown to induce a strong cellular and humoral immune response through activation of DCs and antigen-specific T-cell proliferation. Furthermore, IC31 increases the activation and cytotoxic activity of CD8+ T cells [21].

In this study we compared different vaccination regimens using NYVAC-CN54 and recombinant Env-CN54 protein, in combination with alum alone, with IC31 alone, or with IC31 and alum together. We evaluated induction of Env-specific antibodies, CD4+ and CD8+ T-cell effector function, and the formation of mucosal central and effector memory T-cell subsets. Our findings indicate that IC31 greatly improves vaccination immunogenicity in terms of the development of the Env-specific response.

## Materials and Methods

### Mice

Female BALB/c mice, 5 to 6 weeks old, were purchased from Harlan Laboratory (Indianapolis, IN) and maintained in the Fred Hutchinson Cancer Research Center (FHCRC) animal health resource facility under pathogen-free conditions.

### Ethics statement

All experiments were approved by the FHCRC institutional animal care and use committee. The Office of Laboratory Animal Welfare has approved the FHCRC's Animal Welfare Assurance (#A3226-01).

### Reagents

Purified recombinant HIV-1 Env-CN54 gp140 was manufactured by Polymun (Vienna, Austria). IC31® is a proprietary formulation developed by Intercell (Vienna, Austria) and was supplied by the manufacturer. Alhydrogel (alum) was from Brenntag (Frederikssund, Denmark). The development and immunogenicity of NYVAC-CN54 were previously described [8]. Env peptides spanning the complete CN54gp140 protein sequence were synthesized as 15-mers overlapping by 11 amino acids by Biosynthesis (Lewisville, TX), and were pooled sequentially at 40 peptides per pool (Table 1).

**Table 1:** Aminoacid residues included in each of the four peptide pools used for *in vitro* stimulations, and their location in the Env protein structure.

Pool	AA position	Env structure
1	31–201	V1–V2
2	191–361	V3
3	351–517	V4–V5
4	507–665	gp41

### Vaccine regimens and injections

**Figure 1:**
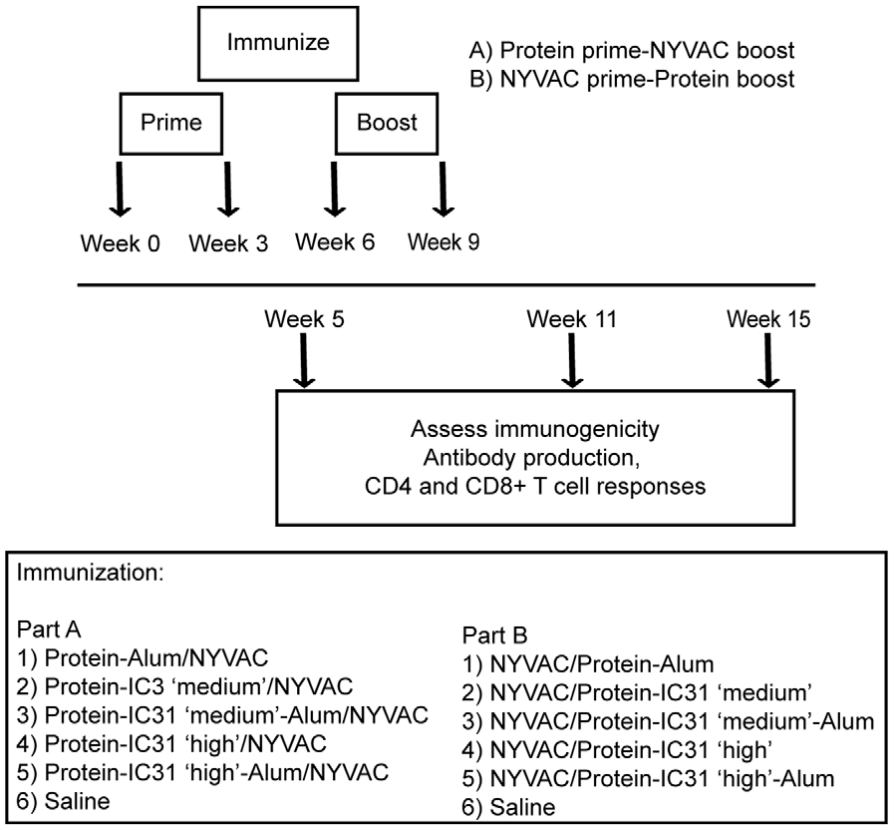
Schematic representation of the vaccination protocol. Three to five mice for each timepoint were injected at weeks 0 and 3 with the prime and at weeks 6 and 9 with the boost. Mice were either injected with saline (negative control, three mice per group), with protein plus alum alone, with protein plus a medium or high dose of IC31 alone, with protein plus alum and a medium or high dose of IC31, or with NYVAC-CN54 (five mice per each group per timepoint). At week 5, 11 or 15, a subset of mice were sacrificed and antibody and T-cell responses were analyzed *ex vivo*.

Mice were divided into 10 test cohorts of 15 mice each, except for the two groups that received protein adjuvanted with alum only, where nine mice each were used. Five cohorts received NYVAC-CN54 as prime and CN54 protein as boost, while the other five cohorts received CN54 protein as prime and NYVAC-CN54 as boost. As a placebo control, two additional cohorts of nine mice each received saline alone. For all test cohorts NYVAC-CN54 was administered at 10^7^ PFU per injection; Env-CN54 was given at 10 µg/mouse per injection. For Env-CN54 adjuvanted with alum alone or with alum and IC31 together, protein was mixed with alum (20 µg) for 1 hour at room temperature with gentle agitation in a Tris buffered solution (20 mM Tris, 150 mM NaCl, pH 7.5). Where indicated, IC31, at a concentration of 1.4 nmol ODN1a (“IC31 medium”) or 4 nmol ODN1a (“IC31 high”), was then added to the protein with alum. For protein adjuvanted with IC31 alone, the same concentrations were used. Mice were intramuscularly injected in the rear quadriceps using a 28-gauge insulin syringe. A schematic representation of the injection schedules and regimens is shown in Figure 1.

### Detection of HIV-1 Env-specific antibodies

Serum was obtained from mice at weeks 5, 11 and 15 and ELISAs were performed following standard procedures. 2HB plates (96-well; Nalge Nunc, Rochester, NY) were coated overnight with 0.9 mg/ml HIV Env-CN54 protein, then washed with buffer (150 mM NaCl, 0.1% Tween 20 in water), blocked for 1 h in 5% nonfat milk with 3% heat-inactivated goat serum and 0.2% Tween 20 in PBS, and then washed again. The serum samples were serially diluted in blocking buffer, then added to the wells in duplicate and incubated for 2 h at 37°C. The plates were then washed, and HRP-conjugated anti-mouse IgG (Thermo Scientific, Waltham, MA), anti-mouse IgG1-HRP, or anti-mouse IgG2a-HRP (both from SouthernBiotech, Birmingham, AL) was added at 1∶2500 dilution and incubated for 1 h at 37°C. After washing, the plates were developed with soluble 3,3′-5,5′-tetramethylbenzene (BM blue POD substrate; Roche Applied Science, Indianapolis, IN); the reaction was stopped by addition of 1 M H_2_SO_4_ and the optical density (OD) was read at 450 nm using a SpectraMax M2 plate reader (Molecular Devices, Sunnyvale, CA). The reported titers correspond to the reciprocal of the highest serum dilution showing a four-times higher OD value than background.

### Intracellular cytokine staining and measurement of cellular proliferation by CFSE dilution

Fresh cells from individual spleens or MLN were stimulated for 5 h with one pool of Env-CN54 peptides, with 0.08% DMSO (peptide diluent) alone as a negative control, or with 1 µg/ml of anti-CD3 and 0.5 µg/ml anti-CD28 as a positive control, adding 10 µg/ml Brefeldin A (eBioscience, San Diego, CA) during the incubation. The cells were then washed, blocked for 10 min with 0.5 µg/ml anti-CD16/CD32 in PBS containing 2% FBS and then the surface staining was performed. The cells were then washed and fixed with fixation permeabilization buffer (eBioscience) for 30 minutes at 4°C. The intracellular staining was then performed and the samples were analyzed on a BD LSRII system (BD Biosciences, Franklin Lakes, NJ). FACS data were analyzed using FlowJo software (TreeStar, Ashland, OR). All antibodies used for stimulation, sorting and staining were obtained from eBioscience, and included anti-CD4 (clone GK1.5), anti-CD8 (clone 53-6.7), anti-CD127 (clone A7R34), anti-KLRG1 (clone 2F1), anti-CD62L (clone MEL-14), anti-IFN-γ (clone XMG1.2), anti-TNF-α (clone MP6-XT22), anti-CD3 (clone 145-2C11), anti-CD28 (clone 37.51) and anti-CD16/CD32 (clone 93).

To perform the cellular proliferation assay, 1×10^6^ splenocytes from each mouse were resuspended in PBS containing 0.1% BSA. Carboxyfluorescein succinimidyl ester (CFSE; Invitrogen, Grand Island, NY) was added to reach a final concentration of 1 µM, and the cells were incubated at room temperature for 5 minutes. Culture medium containing 10% FBS was then added and the cells were incubated for 5 minutes on ice, washed twice and then plated at a concentration of 10^5^ cells/well in the presence of Env-CN54 peptide pool 1, DMSO alone, or anti-CD3/CD28 at the same concentrations as used for the intracellular cytokine staining stimulation. After three days of culture, cells were stained following the intracellular cytokine staining protocol. Proliferation was measured as the percentage of CFSE-low cells.

### Statistical analyses

Data were analyzed by two-tailed unpaired Student's *t* tests using PRISM 5 software (GraphPad Software, La Jolla, CA). Welch's correction was applied for non-equally distributed variances.

## Results

### All vaccinations elicited a humoral response to Env

**Figure 2:**
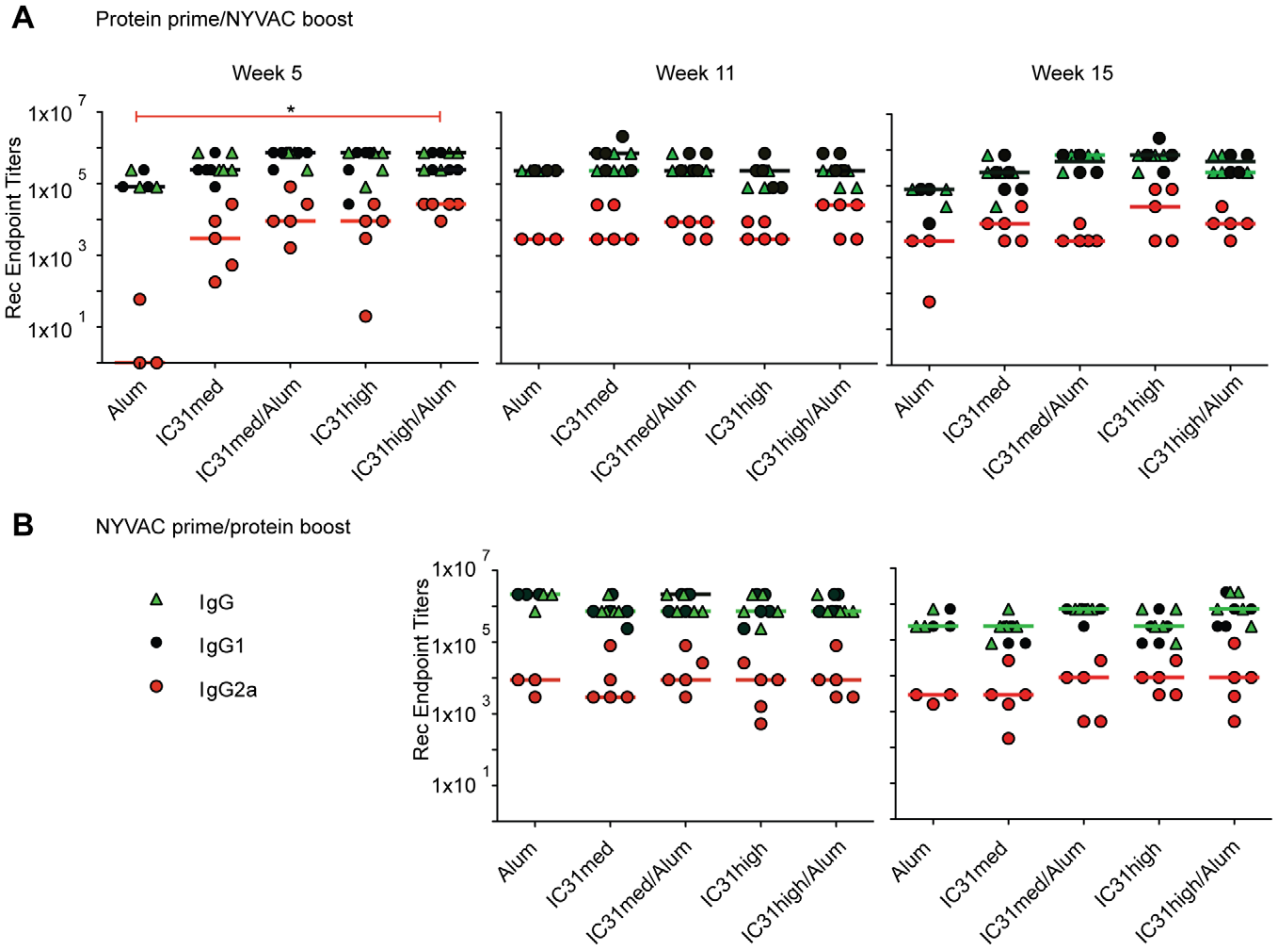
Robust antibody responses are induced by all vaccination regimens. Serum levels of total IgG (green triangles), IgG1 (black circles) and IgG2a (red circles) were measured at weeks 5, 11 and 15. A) Mice were primed at weeks 0 and 3 with Env-CN54 gp140 with alum alone, or a medium (“med”) or high concentration of IC31 with or without alum, and boosted at week 6 and 9 with NYVAC-CN54. B) Mice were primed at weeks 0 and 3 with NYVAC-CN54 and boosted at week 6 and 9 with Env-CN54 gp140 protein in combination with the adjuvants as indicated. Total IgG could not be detected in the control mice. The graphs show the antibody titers detected by ELISA at weeks 5, 11, and 15. Bars correspond to the medians. *p<0.05.

We first examined the Env-specific antibody response following administration of various vaccine regimens (Fig. 1). After priming with protein, the groups receiving Env-IC31 induced at least a three log increase in their IgG2a titer at week 5 as compared to Env-alum alone (Fig. 2A). This difference was statistically significant when the high dose of IC31 plus alum was used, where a seven log-difference was achieved (p<0.05). We observed a similar trend for total IgG and IgG1. After boosting with NYVAC-CN54, serum IgG2a titers at week 11 trended slightly higher in the groups that were primed with protein adjuvanted with IC31 plus alum as compared to protein with either adjuvant alone, although the differences were not significant. At week 15 we did not observe any significant differences between the groups (Fig. 2A).

Among mice primed with NYVAC-CN54 and boosted with protein plus adjuvant, there were no differences in antibody induction at weeks 11 or 15, regardless of adjuvant (Fig. 2B).

### Env-specific CD4+ T-cell responses are increased in magnitude and durability by IC31-protein prime/NYVAC-CN54 boost

**Figure 3:**
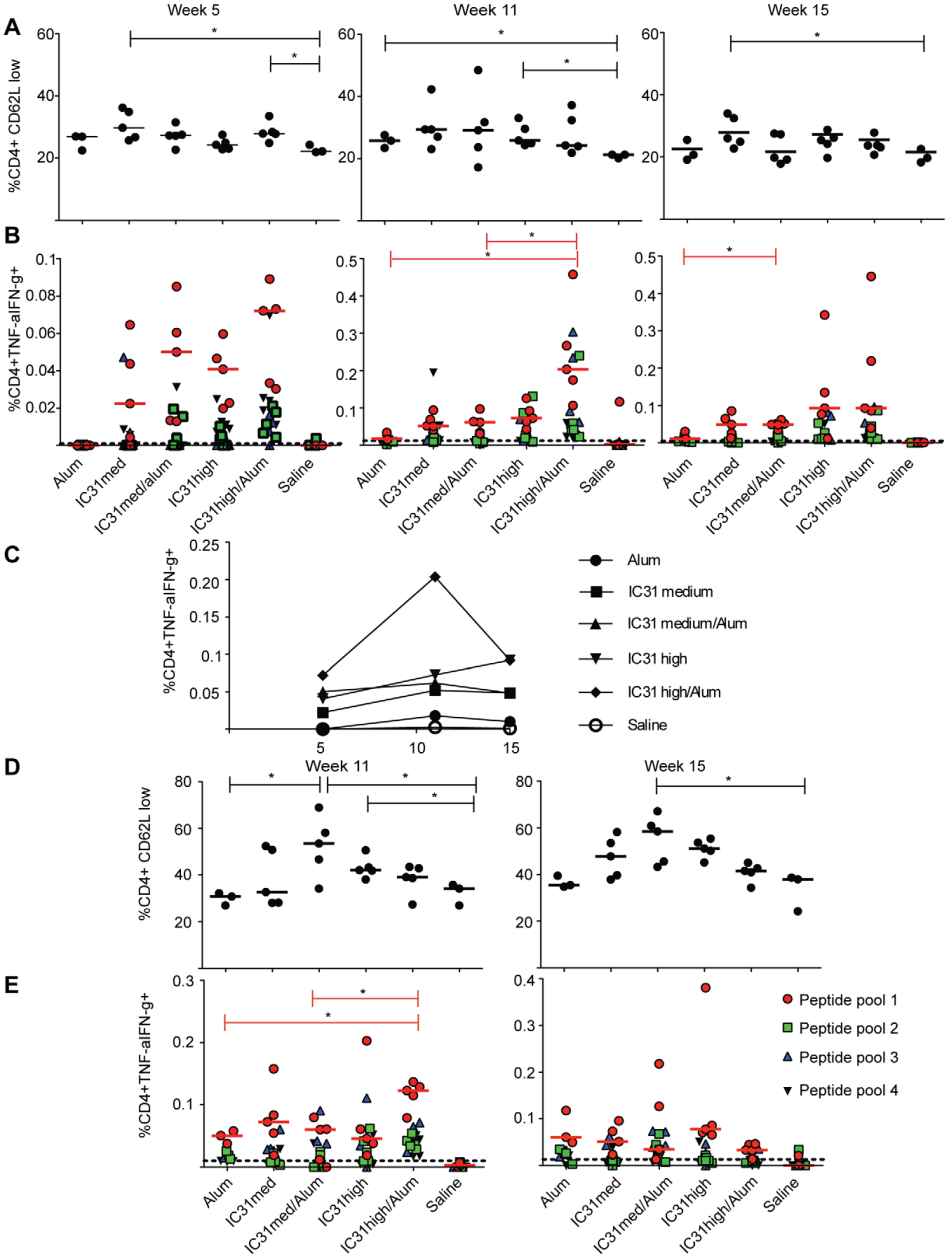
Durable Env-CN54 specific CD4+ T-cell responses are detected following vaccination when Env/alum/IC31 is used as a prime. (A–C) Mice were primed with Env-CN54 gp140 in combination with either alum alone or a medium (“med”) or high concentration of IC31 with or without alum and then boosted with NYVAC-CN54 at three week intervals. A) Frequencies of activated CD4+ T cells as indicated by low CD62L expression at weeks 5, 11 and 15. Each data point corresponds to a single mouse in the experimental group. B) Percentage of CD4 T cells secreting cytokines (TNF-α and IFN-γ) upon stimulation with four peptide pools covering the full length of Env-CN54 gp140, as determined by ICS. C) Kinetic analysis of the frequencies of cytokine secreting CD4+ T cells following vaccination. Data points correspond to the medians of the frequencies obtained by stimulation with peptide pool 1. D–E) Mice were primed with NYVAC-CN54 and boosted with Env-CN54 gp140 in combination with adjuvants as listed. (D) Frequencies of activated CD4+ T cells as indicated by low CD62L expression at weeks 11 and 15. Each data point corresponds to a single mouse in the experimental group. (E) Percentage of CD4+ T cells secreting cytokines at weeks 11 and 15. For B and E, data points following stimulation with peptide pool 1 (red circles), pool 2 (green squares), pool 3 (green triangles) and pool 4 (black triangles) are distinguished, while the dotted line corresponds to the average obtained upon DMSO stimulation. Bars correspond to median values (medians for peptide pool 1 stimulation only in B and E). *p<0.05.

Next we examined CD4+ T-cell responses two weeks after protein prime (week 5) and two and six weeks after boosting with NYVAC-CN54 (Fig. 1). We used decreased expression of CD62L to indicate general T-cell activation, and observed a significant increase in CD4+ T-cell activation at week 5 when the medium dose of IC31 alone or the high dose of IC31 plus alum was used in combination with protein versus the saline control (p<0.05 each; Fig. 3A). Although the differences were not significant in all groups, there was also a general trend for increased CD4+ T-cell activation following the NYVAC-CN54 boost versus the saline control at weeks 11 and 15 (Fig. 3A).

We also evaluated CD4+ T-cell function by measuring cytokine secretion upon *in vitro* stimulation. Priming with Env-CN54 adjuvanted with IC31 at any dose, with or without alum, induced a higher percentage of cytokine producing cells at week 5 as compared to the saline control or to priming with Env-CN54 adjuvanted with alum alone (Fig. 3B). The median frequency of cytokine-producing CD4+ T cells detected after stimulation with peptide pool 1, which induced the majority of detectable responses, ranged from 0.023% when the adjuvant was the medium dose of IC31 alone, to 0.072% with the high dose of IC31 plus alum. Boosting with NYVAC-CN54 induced a two- to three-fold increase in the CD4+ T-cell response in all regimens. Moreover, mice immunized with the high dose of IC31 plus alum had a significantly higher frequency of cytokine-producing CD4+ T cells versus those receiving the medium dose plus alum or alum alone (0.204% for IC31 high/alum vs. 0.062% for IC31 medium/alum and 0.018% for alum alone, p = 0.016 and p = 0.033, respectively; Fig. 3B). Six weeks after the final immunization (week 15), we again observed that inclusion of IC31 with the protein prime induced a CD4+ T-cell response of greater magnitude than inclusion of alum alone (Fig. 3B). The CD4+ T-cell response to peptide pool 1 is shown longitudinally in Fig. 3C, demonstrating that adjuvanting the protein prime with the high dose of IC31, alone or with alum, induced the greatest expansion of Env-specific CD4+ T cells and this population was still present at week 15.

When the regimens were reversed, with NYVAC-CN54 as prime and the protein-adjuvant combinations as boost, we observed a general trend for increased T-cell activation when the adjuvant included IC31 (Fig. 3D). CD4+ T-cell responses were of lower magnitude at week 11 as compared to protein prime/NYVAC-CN54 boost. We could still detect a slightly superior adjuvant activity with the high dose of IC31 plus alum, although this response was only significantly different from alum alone and the medium dose of IC31 plus alum (p<0.05 each; Fig. 3E). At week 15, we detected a very low percentage of Env-specific CD4+ T cells secreting cytokines, and there were no significant differences between groups (Fig. 3E).

### Protein/IC31 adjuvant prime, NYVAC-CN54 boost regimens induce superior Env-specific CD8+ T-cell responses

**Figure 4:**
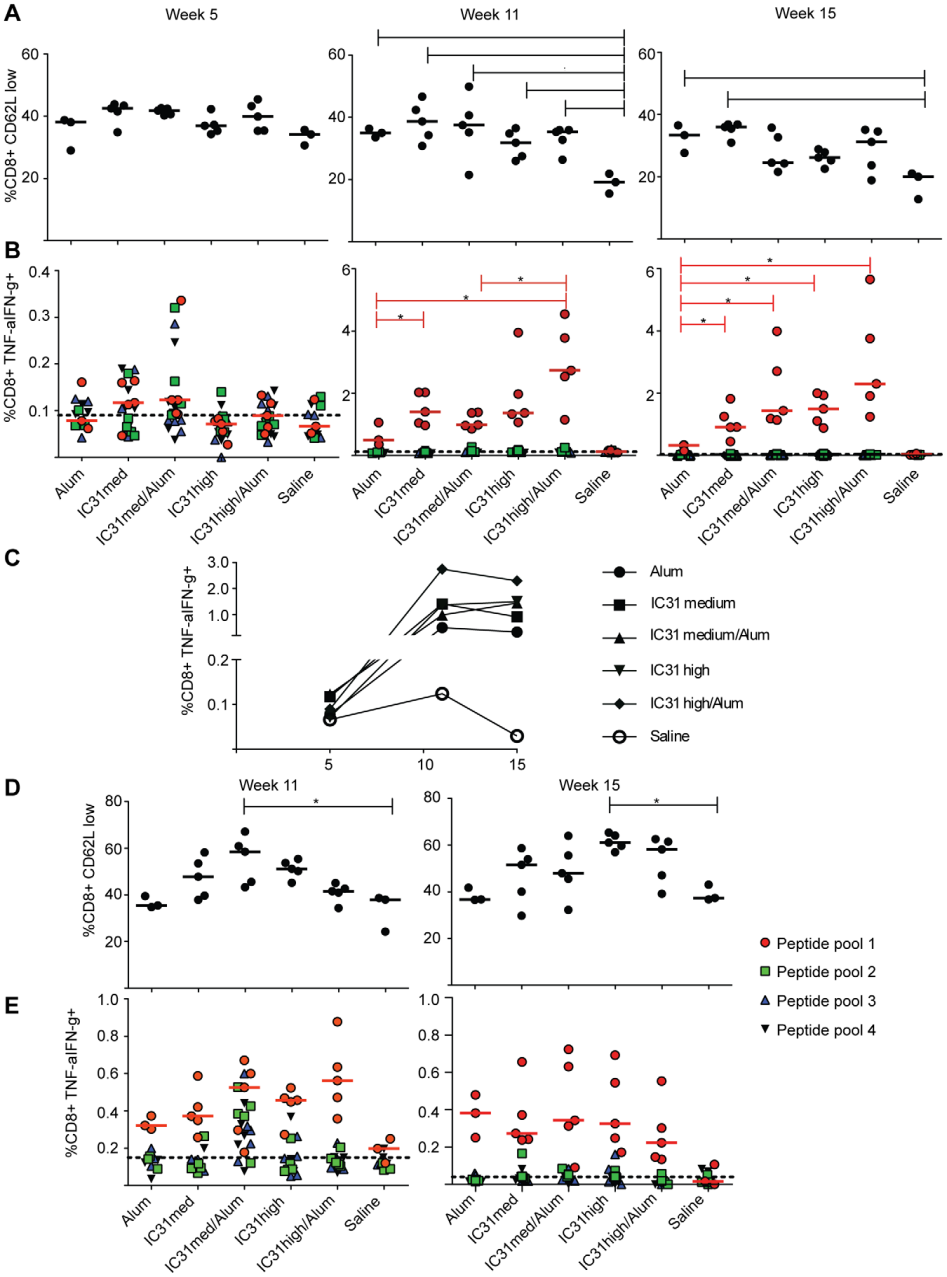
Durable Env-CN54 gp140 specific CD8+ T-cell activation and cytokine secretion are detected following vaccination. (A–C) Mice were primed with Env-CN54 gp140 in combination with alum alone, or with a medium (“med”) or high concentration of IC31 with or without alum, and then boosted with NYVAC-CN54 at three week intervals. A) Frequencies of activated CD8+ T cells as indicated by low CD62L expression at weeks 5, 11 and 15. Each data point corresponds to a single mouse in the experimental group. B) Percentage of CD8+ T cells secreting cytokines upon stimulation with the four peptide pools, as determined by ICS. C) Kinetic analysis of the frequencies of cytokine secreting CD8+ T cells following vaccination. Data points correspond to the medians of the frequencies obtained by peptide pool 1 stimulation. D–E) Mice were primed with NYVAC-CN54 and boosted with Env-CN54 gp140 in combination with adjuvants as indicated. D) Frequencies of activated CD8+ T cells as indicated by low CD62L expression at weeks 11 and 15. Each data point corresponds to a single mouse in the experimental group. E) Percentage of CD8+ T cells secreting cytokines at weeks 11 and 15. Labels are as shown in Figure 3. *p<0.05; **p<0.01.

We next evaluated the effect of the different regimens on the activation status and cytokine production of CD8+ T cells. As a marker of CD8+ T-cell activation we again utilized decreased expression of CD62L. Two weeks after priming with protein/adjuvant, there was a trend toward an increase in the percentage of activated CD8+ T cells for all vaccine groups versus the saline control (Fig. 4A). Two weeks after NYVAC-CN54 boost, we detected a significant increase in CD8+ T-cell activation in all vaccine groups versus the saline control (p<0.01 for alum only and IC31 medium plus alum, p<0.05 for all others; Fig. 4A). At week 15, although the trend was the same, a significant difference versus saline was observed only in the groups primed with protein and alum alone or protein with the medium dose of IC31 alone (p<0.05 each; Fig. 4A).

We next examined the effect of the different regimens on CD8+ T-cell cytokine production. We did not detect significant antigen-specific CD8+ T-cell responses in any group following the protein/adjuvant primes (Fig. 4B). After boosting, we detected cytokine secretion in all groups, although there were significant differences in frequencies between groups (Fig. 4B), with the greatest difference observed between protein prime with the high dose of IC31 plus alum versus protein prime with alum alone (2.74% median frequency of response to peptide pool 1 versus 0.5%; Fig. 4C). At week 15, we also observed a robust Env-specific effector response in the CD8+ T-cell compartment in all groups primed with protein and IC31, while the group primed with protein and alum alone had a significantly lower response rate (p<0.05 each; Fig. 4B). In summary, priming with protein and the high dose of IC31 plus alum generated an Env-specific CD8+ T-cell response with the highest magnitude and durability (Fig. 4C).

When the regimens were reversed, with NYVAC-CN54 prime and the protein-adjuvant combinations as boost, CD8+ T-cell activation followed a trend similar to that observed with the protein prime/NYVAC-CN54 boost regimens (Fig. 4D). Following protein boost, the groups for which IC31 was used as an adjuvant showed an increase in CD8+ T-cell activation as compared to alum alone or the saline control. At the memory timepoint, we observed a trend toward increased CD8+ T-cell activation in the groups boosted with protein and IC31 compared to protein with alum alone or the saline control, although this only reached significance for the high dose of IC31 alone versus the saline control (p<0.05; Fig. 4D). While all adjuvant/protein formulations elicited an elevated frequency of CD8+ T-cell response over the saline control, none reached statistical significance either directly after boosting or at the memory timepoint (Fig. 4E).

### IC31 promotes an enhanced memory response

**Figure 5:**
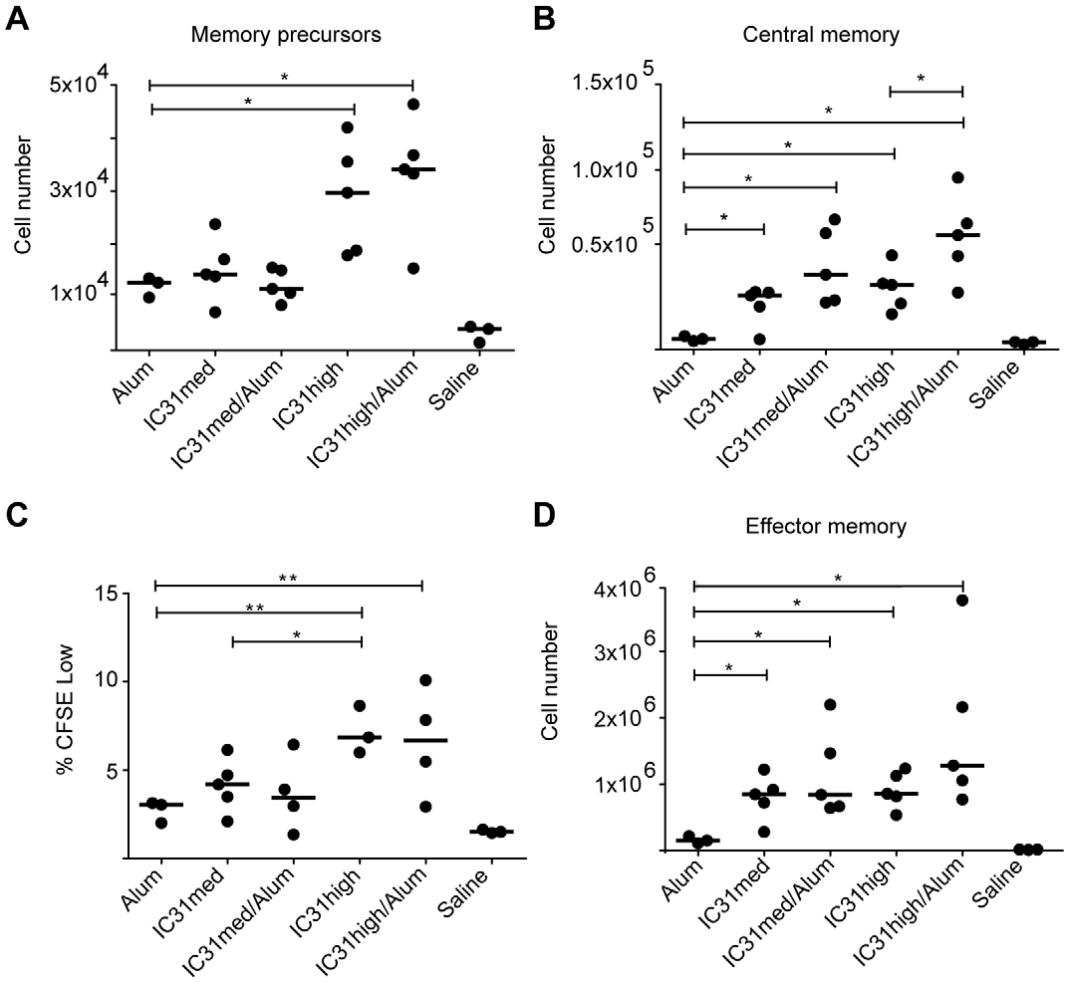
The memory T-cell pool is enhanced by Env/IC31 prime. A) At week 11, the numbers of CD8+ memory precursor cells (IFN-γ+ and/or TNF-α+ CD8+ KLRG1-low CD127+) in the spleen were determined for mice that were primed with Env-CN54 gp140 with various adjuvants as indicated and boosted with NYVAC-CN54. The graph shows the results obtained after *in vitro* stimulation with peptide pool 1. B) At week 15, memory T-cell numbers were assessed in the spleen. Central memory T cells were defined as CD8+ T cells secreting IFN-γ and/or TNF-α with high expression of CD62L. The graph depicts central memory cell numbers induced by the various vaccination regimens following *in vitro* re-stimulation with peptide pool 1. C) Proliferation of spleen CD8+ T cells from week 15 was determined by CFSE dilution. The graph shows the frequency of proliferating CD8+ T cells following *in vitro* stimulation with peptide pool 1 for 3 days. D) Effector memory T cells from week 15 were defined as CD8+ T cells secreting IFN-γ and/or TNF-α, expressing low levels of CD62L. The graph shows the values obtained after *in vitro* stimulation with peptide pool 1. Bars indicate the median value. *p<0.05 and **p<0.01. Each data point corresponds to a single mouse in the experimental group.

Since priming with protein adjuvanted with IC31 induced CD4+ and CD8+ T-cell responses that persisted, we further investigated the ability of this regimen to induce immunological memory. We first determined the number of memory precursors two weeks after NYVAC-CN54 boost by measuring the number of antigen-specific CD8+ T cells (producing TNF-α and/or IFN-γ following stimulation with peptide pool 1) that were CD127+KLRG1- [22]. Mice primed with protein adjuvanted with the high dose of IC31, with or without alum, had significantly more memory precursors (p<0.05 each vs. alum only, Fig. 5A), demonstrating that IC31 is superior to alum alone in terms of inducing CD8+ T-cell memory precursors.

We next determined the number of central memory (T_CM_) and effector memory (T_EM_) cells six weeks after boost by distinguishing Env-specific CD8+ T cells by cytokine expression and further gating on CD62L positive or negative cells, respectively [22]. The greatest expansion of the central memory cell compartment occurred when mice were primed with protein adjuvanted with the high dose of IC31 plus alum (Fig. 5B). In all cases, adjuvanting with IC31 resulted in significantly higher expansion than with alum alone (p<0.05 each; Fig. 5B). Additionally, CD8+ T cells at the memory timepoint had significantly increased proliferative capacity when the high dose of IC31 was used, with or without alum (p<0.01 each vs. alum only; Fig. 5C). This is consistent with previous studies demonstrating that T_CM_ cells are superior to T_EM_ cells in proliferation [23], [24]. Finally, we found that a protein prime including IC31, with or without alum, resulted in significantly increased effector memory cell generation (p<0.05 each vs. alum only; Fig. 5D).

### Env-specific CD8+ T cells are induced in the mesenteric lymph nodes when the protein prime includes IC31

**Figure 6:**
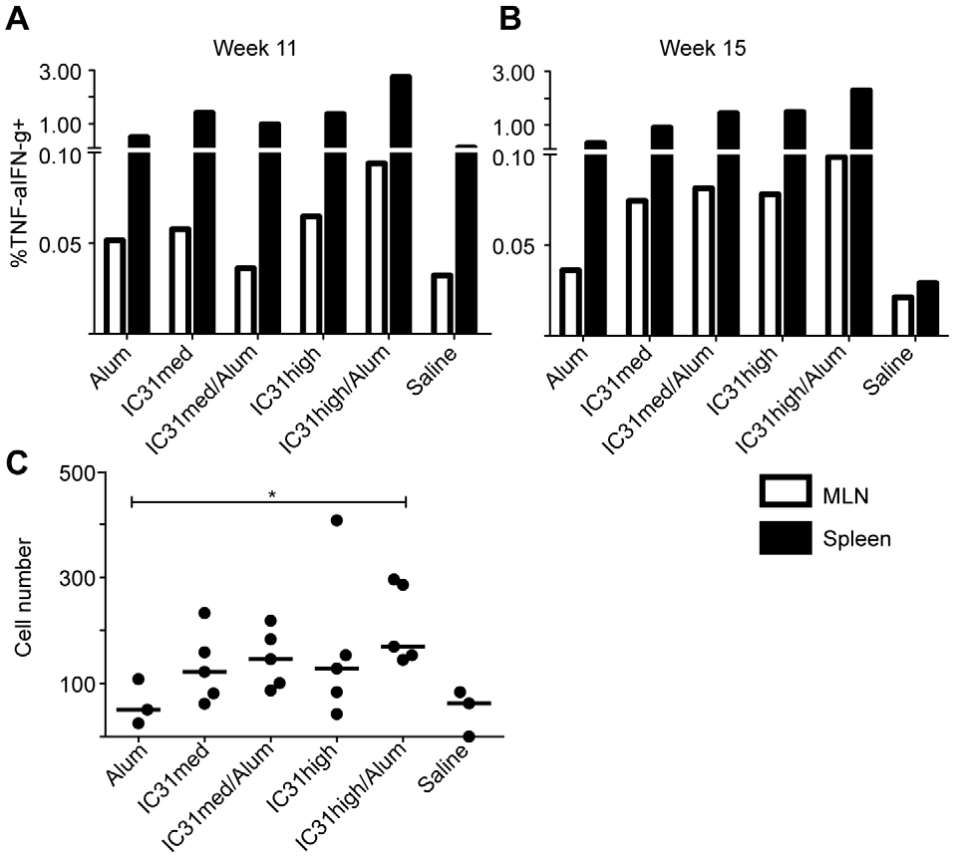
Detection of Env-CN54 gp140 specific CD8+ T cells in the GALT of vaccinated mice. The frequencies of CD8+ T cells secreting cytokines in response to CN54 peptide pools were evaluated in the mesenteric lymph nodes (MLN) of vaccinated mice at week 11 (A) and at week 15 (B). Graphs show the median CD8+ T-cell responses from the MLN (white bars) and from spleen (black bars) following stimulation with peptide pool 1. C) Effector memory T-cell numbers within the MLN were determined as described for Figure 5. Each data point corresponds to a single mouse in the experimental group.

We next examined the Env-specific CD8+ T-cell response in the mesenteric lymph nodes (MLN) following the protein prime/NYVAC-CN54 boost vaccination regimens. Both immediately following NYVAC-CN54 boost and at a memory timepoint there were cytokine-secreting CD8+ T cells present in the MLN, regardless of the adjuvant used. The percentages were similar at the two timepoints and the pattern was comparable to that observed in the spleens (Fig. 6A–B). Specifically, at week 11, the high dose of IC31 plus alum induced the highest percentage of Env-specific CD8+ T cells (0.094%, compared to 0.065% for IC31 high alone, 0.058% for IC31 medium alone, 0.036% for IC31 medium plus alum and 0.052% for alum alone). Six weeks after boosting with NYVAC-CN54 we detected Env-specific T_EM_ in the MLN of mice that received the protein prime adjuvanted with the high dose of IC31 plus alum. The number of T_EM_ in these MLN was significantly higher than in mice primed with protein and alum alone, where Env-specific T_EM_ were virtually undetectable (p<0.05; Fig. 6C). We could not detect Env-specific CD4+ T cells in the MLN of mice from any group (data not shown), despite detecting a potent response in the spleen (Fig. 3).

## Discussion

In our study, we tested the novel adjuvant IC31 in combination with recombinant Env-CN54 protein in a heterologous prime-boost vaccination regimen with a NYVAC-CN54 vector encoding for the same Env variant. To our knowledge, this is the first study utilizing IC31 for an HIV vaccine immunogenicity study. IC31 was previously shown to induce Th1-like antibody immunity and to facilitate isotype switching to IgG2a [25], [26]. In the context of HIV vaccines, this characteristic is particularly valuable, since the importance of antibody functional activities other than neutralization, such as ADCC, has recently been highlighted by the correlates analysis of the RV144 trial [27]. In our study, we observed that adjuvanting a protein prime with IC31 induced increased levels of IgG2a and lowered the ratio of IgG1/IgG2a as compared to priming with protein plus alum alone. We also showed that NYVAC-CN54 prime followed by protein boost induced an elevated level of IgG2a antibodies, regardless of the adjuvant used (Fig. 2), confirming that NYVAC-CN54 is an excellent prime for antibody induction, as previously reported [9].

Isotype switching to IgG2a has been shown to be dependent on production of IFN-γ [28]. While CD4+ T-cell activation was increased following protein prime at weeks 5 and 11 regardless of the adjuvant used, inclusion of IC31 maintained this activation at a later timepoint (Fig. 3A). Induction of a CD4+ T-cell response is essential to prevent the exhaustion of CD8+ memory T cells upon re-challenge [29]. This is particularly important when considering the vaccination protocols often used for HIV vaccine trials, which utilize multiple challenges with the same antigen, thus potentially leading to exhaustion of CD8+ T cells in the absence of antigen-specific CD4+ T cells. We hypothesize that the inclusion of IC31 prevented this exhaustion by maintaining CD4+ T-cell activation (Fig. 4B). Importantly, the responses that we detected for both CD4+ and CD8+ T cells were mostly induced by Env peptide pool 1, which includes sequences from the V1/V2 loops. Antibody responses to the same region of similar Env proteins have been recognized as one of the principal correlates of risk, associated with a 43% reduction in infection in the RV144 trial vaccine recipients [27].

Finally, the inclusion of IC31 in a protein prime induced a higher number of Env-specific CD8+ T cells in the MLN (Fig. 6), while we did not detect any antigen-specific CD4+ T-cell response at this site. This is likely to be advantageous for an HIV vaccine, since it will not increase the frequency of activated CD4+ T cells, which are potential targets of the virus, at an important site of replication. Moreover, the induction of CD8+ T-cell memory at a mucosal site, and specifically in the gut, is likely to be important in preventing HIV replication early after infection. This effect on mucosal immunity might be one of the causes for the protection from *Chlamydia* infection in a recent vaccination study where IC31 was used as the adjuvant [30].

Our results can likely be explained by the capacity of IC31 to signal through TLR9/MyD88 and induce dendritic cell maturation, which is thought to then be responsible for driving Th1 responses [30]. The different cellular distribution of TLR9 in mice and humans has historically posed a problem with results gained from mouse models not translating into humans [31]. Importantly, however, IC31 has been administered in two clinical trials of tuberculosis vaccines in naïve as well as in BCG-vaccinated and TB-positive subjects, where it was shown to induce a Th1 immune response with IFN-γ secretion by PBMC [32]. Thus, we expect IC31 to have a similar potent effect when included in an HIV vaccine administered to humans.

Our study also demonstrates the advantages of combining two distinct adjuvants, namely IC31 and alum, for inducing both T-cell and antibody responses. IC31 has been shown to induce strong type 1 immune response [21], while alum is known to induce type 2 immune responses [33].

In this vaccination schedule, the order of NYVAC-CN54 and protein is important for the induction of a strong cellular response. The efficacy of NYVAC-CN54 as a boost was reported in a recent NHP study [34]. Our study likewise found that NYVAC-CN54 provided an excellent boost in terms of CD4+ and CD8+ T-cell activation and function.

### Conclusions

Our study reveals multiple innovative findings for the field of HIV vaccinology. First, we showed that IC31 used in combination with Env-CN54 in the priming phase of a vaccination regimen induces isotype switching and the production of Env-specific IgG2a. IC31 also increases the magnitude of CD4+ and CD8+ Env-specific T-cell responses, the development of a central memory and effector memory compartment and the presence and persistence of antigen-specific T cells in gut-associated lymphoid tissues. Second, we demonstrated that priming with NYVAC-CN54 followed by protein/adjuvant boost does not induce B and T-cell immunity to the same extent as the reverse vaccination strategy. Taken together, our study supports the use of IC31 as a protein adjuvant for future HIV vaccine trials, in combination with a viral vector boost, such as NYVAC-CN54.
